# On the optimal layout of (*K*_*p*_ − *C*_*p*_)^*n*^ into grid and certain structures

**DOI:** 10.1038/s41598-025-98962-5

**Published:** 2025-05-16

**Authors:** G. Caroline Vincy, David Raj Micheal

**Affiliations:** Department of Mathematics, School of Advanced Sciences, Vellore Institute of Technology, Chennai, Tamil Nadu 600127 India

**Keywords:** Wirelength, Maximum subgraph problem, Graph embedding, Optimality, Engineering, Mathematics and computing

## Abstract

Interconnection networks constitute complex configurations of processors and communication links that facilitate data transmission between processors in a parallel computing system. Their architecture and design heavily depend on parameters such as wirelength, dilation, bandwidth, and minimum cutwidth. The process of constructing layouts on a board using the necessary modules determines the manufacturing cost in computer networks, where knowledge of graph embedding serves as an integral tool. Placement problems associated with circuit designs, for which no deterministic techniques exist, can be addressed by obtaining the optimal architecture through the embedding function. This article focuses on embedding the guest graph (*K*_*p*_ − *C*_*p*_)^*n*^ into various host graphs, including the grid, generalized book graph, triangular snake, and variants of the banana tree. Furthermore, their optimal wirelengths are also obtained.

## Introduction

The integration of one graph, known as the guest, into another, typically referred to as the host, and the analysis of its attributes are considered essential in fields where computers and processors are widely used. Multiprocessing systems rely on interconnection networks, which are a key area of research. Nearly all computer scientists and engineers recognize that a graph represents the topological characteristics of an interconnection network’s structure. Graph theory has been established as a powerful mathematical tool for designing and understanding interconnection networks.

Very Large Scale Integrated (VLSI) systems are no exception to the widespread use of networks. These systems consist of numerous interdependent subsystems, modules, and an immense number of integrated transistors and wires. To effectively manage and analyze such complex structures, graph theory is critically important.

Graph embedding plays a crucial role in assessing interconnection networks through graph partitioning, algorithms, and graph indices. Its practical applications extend beyond network design in parallel computing and data structures. When evaluating its performance, various criteria are considered to minimize costs, including wirelength, along with other metrics like dilation, edge congestion, load, and expansion. An embedding function that maps the vertices of the guest graph to the host graph evaluates these metrics. Dilation, refers to the longest path in the host graph connecting images of the corresponding vertices from the guest graph and edge congestion, refers to the maximum frequency of an edge in the host graph. Similarly wirelength is an additional distance metric that involves the edge congestion values. Load and expansion define the relationship between the number of vertices in the guest graph and the number of vertices in the host graph. These measures are employed in various models, including biological networks, scheduling problems, VLSI design, and parallel networks. In particular, VLSI design involves several steps, with floorplanning being one key aspect. Floorplanning defines the shape and arrangement of major components within a layout. This initial layout design not only aids in the subsequent placement process but also offers crucial overall insights, such as the dimensions and locations of inputs and outputs. A VLSI circuit is represented as a network of interconnected blocks, with each segment of the circuit organized into blocks. Common evaluation criteria for floorplanning include total wirelength, congestion, and timing criticality. The placement procedure aims to optimize the timing performance of the system. Coordination performed during the arrangement of blocks often sets constraints on specific wirelengths. Congestion describes the ease of performing routing in later stages, with congestion maps commonly used during placement to identify challenging areas and adjust the placement to reduce congestion.

Graph embedding was introduced in 1998 by Harper^1^, thus initiating the field of study. Since then, various structures have been examined and analyzed based on their relevance and importance. Among these, hypercubes and their variants emerged as particularly significant. Hypercube and its wirelength on embedding it in a grid network was computed in^2^. The embedding of circulant networks into grid and the grid into the circulant network^3^, called both-way embedding, where the two graphs taken for study is used as the guest and the host alternatively, computing its dilation and wirelength parameter. For an embedding problem, when the host graph considered is a linear array, is referred to as the minimum linear arrangement problem(MinLA). The MinLA of bijective connection graphs, which includes graphs such as spined cubes, twisted cubes, etc., as subfamilies, is studied in^4^. The 3-ary *n*-cubes is a well-known prominent structure, and its maximum subgraph problem and embedding it into structures like circulant networks, grids, and trees was determined in^5^. The optimal ordering of the hierarchical cubic network is determined and embedded into *k*-rooted complete binary tree in^6^. In recent years, the structure of the *n* Cartesian product of $K_{4}$ was analyzed and its embedding was studied in^7^. The maximum subgraph problem was determined for the structure of hierarchical folded cubes and embedded into the linear array and tree structures by^8^.

The structure $(K_p - C_p)^n$ is generated through the cartesian product of its first dimension, increasing in complexity as the dimension grows. The primary motivation for studying this graph stems from its distinctive structural properties, including regularity, rigidity, recursive construction, and other significant characteristics that contribute to its uniqueness and mathematical importance. Recent work^9^ has addressed the maximum subgraph problem of $(K_{p}-C_{p})^{n}$ for any $n$, where $p$ is an odd number, $n \ge 2$, and $p \ge 9$. However, a gap remains in identifying additional suitable host graphs into which the structure can be embedded using graph embedding techniques and in computing their wirelength.

The main contributions of this study are as follows:

- Employing a graph embedding technique to determine the optimal layout for embedding $(K_{p}-C_{p})^{n}$ into various host graphs, including the grid, generalized book graph, triangular snake, extended banana tree, and arbitrarily fixed generalized banana tree.
- Computing the exact wirelength corresponding to the optimal layout.
- Providing algorithms to determine the optimality of any embedding function $\Psi$ that embeds $(K_{p} - C_{p})^{n}$ into the host graphs listed in (a). Furthermore, using Lemma 2.1, these algorithms also compute the wirelength when $\Psi$ is optimal.
- Analyzing the computational complexity of all proposed algorithms.

The paper is organized into various sections, with section 2 focusing on preliminaries such as basic definitions and theorems, section 3 explains briefly the construction and structural properties of $(K_p - C_p)^n$, and the following sections discusses the embedding function and the wirelength parameter estimated for different host graphs.

## Preliminaries

Suppose that *G* is a graph, and that its vertex and edge sets are *V*(*G*) and *E*(*G*), respectively. The following is an introduction to the prerequisites needed to read the article:

### Definition 1

^1^Consider the finite graphs $G_{g}$ and $G_{h}$. The pair $\Psi = (f,P_{f})$ that represents an embedding $\Psi$ of $G_{g}$ into $G_{h}$ is defined as follows: The map *f* is one-to-one map from the vertex set of $G_{g}$ to the vertex set of $G_{h}$. Let $P_{f}$ be a one-to-one map from $E(G_{g})$ to $\{P_{f}(e): P_{f}(e)$ is a path in $G_{h}$ between *f*(*u*) and *f*(*v*) for $e=(uv) \in E(G_{g})$ }.

The Maximum Subgraph Problem is NP-complete^10^ and may be formulated in two different ways, depending on how we count the edges^11^. They are the two variants stated below:

### Definition 2

^12^Suppose that *N* is a network and that $N^{'}$ is a subset of the vertex set of *N*. Consider $I_{N}(N^{'})$, a subset of the edge set of *N*, where both of the vertices belong to the set $N^{'}$ and their end vertices are $(n_{1},n_{2})$. Let $I_{N}(k)$ be a maximum size of $I_{N}(N^{'})$, including all subsets $N^{'}$ of size *k*.

### Definition 3

^12^For a network $N$, the set $\theta _N(N')$ is defined as the set of all edges $(n_1, n_2)$ in $E(N)$ where the first vertex $n_1$ belongs to a subset $N' \subseteq V(N)$, and the second vertex $n_2$ does not belong to $N'$. Let $k$ be a positive integer, $\theta _N(k)$ is the minimum size of $\theta _N(N')$, where the minimum is taken over all subsets $N' \subseteq V(N)$ of size $k$.

The alternative name for the Maximum subgraph problem is Edge isoperimetric problem (*EIP*) as in Definition 2 of counting the edges. The EIP aims to identify a set $N^{'}$ with $|N^{'}| = n$, $n \in \{1, 2, \dots ,n\}$. For a $r-$regular, the two EIP types mentioned above have the following relation

$$2I_{N}(k) + \theta _{N}(k) = rk$$.

### Definition 4

^13^An embedding of $G_g$ into $G_h$ is defined by a mapping $\Psi$. For any edge $e$ in $G_h$, the value $EC_{\Psi }(e)$ quantifies the number of edges $(u, v)$ in $G_g$ such that the edge $e$ is part of the path $P_{\Psi }(u, v)$ connecting $\Psi (u)$ and $\Psi (v)$ within $G_h$.

The edge congestion of the embedding $\Psi$ is given by $EC_{\Psi }(G_g, G_h) = \max _{e \in E(G_h)} EC_{\Psi }(e)$, which is the highest $EC_{\Psi }(e)$ value among all edges $e$ in $G_h$. The overall edge congestion for embedding $G_g$ into $G_h$, denoted $EC(G_g, G_h)$, is defined as $EC(G_g, G_h) = \min _{\Psi : G_g \rightarrow G_h} EC_{\Psi }(G_g, G_h)$. This represents the smallest possible edge congestion across all embeddings of $G_g$ into $G_h$.

### Definition 5

^13^The wirelength of an embedding $\Psi$ of $G_g$ into $G_h$ is defined as the total sum of edge congestions across all edges in $G_h$. The overall wirelength of the embedding is obtained by finding the minimum wirelength over all possible embeddings $\Psi$ of $G_g$ into $G_h$. Formally, the optimal wirelength is expressed as $WL(G_g, G_h) = \min _{\Psi : G_g \rightarrow G_h} WL_{\Psi }(G_g, G_h)$.

### Lemma 2.1

^13^*Consider an arbitrary graph *$G_{g}$ *that has been embedded into *$G_{h}$ *by* $\Psi$.*Let* *S be a partition that separates the graph* $G_{h}$*, such that* $G_{h}$ *is divided into two components* $G_{h1}$ *and* $G_{h2}$ *by removing the edges of* *S. Now, let* $G_{g1} = \Psi ^{-1}(G_{h1})$ *and* $G_{g2} = \Psi ^{-1}(G_{h2})$*. Moreover, S meets the conditions listed below:*

1. *For every edge in either of the components* $$E(G_{gi}),$$$$i=1,2,$$*S has no edges in the path *$$P_{f}$$* that maps the edges of *$$G_{gi}$$ *to their respective*$$G_{hi}$$.
2. *The set S has exactly only one edge *(*a, b) by* $$P_{f}(a,b),$$$$(a,b) \in E(G_{g})$$ *with a belonging to *$$V(G_{g1})$$ *and b belonging to *$$V(G_{g2})$$.
3. *Sets *$V(G_{g1})$ *and* $V(G_{g2})$* are optimal. In other words, *$E(G_{g1})$* and* $E(G_{g2})$* are the subgraphs that is induced with the maximum number of edges of *$G_{g}$*, with *$|V(G_{g1})|$* and* $|V(G_{g2})|$*, appropriately*.

*The value, *$EC_{\Psi }$* represents the smallest value obtained when considering all mappings *$\Psi$*, with the specific calculation for a subset *$S$* determined by summing the degrees of vertices in either of the components *$G_{gi}$*, subtracted by twice the number of edges in* $G_{gi}$*, for* $i = 1,2$.

### Lemma 2.2

^13^*For the embedding function *$\Psi$*, let *$[vE(G_h)]$* denote a multiset of edges from *$G_h$*, where each edge *$e$* is repeated exactly *$v$* times. Furthermore, let *$R_j$* represent an edge cut of *$G_h$ that satisfies the conditions outlined in Lemma 2.1. *Assuming*$R_j$* is an edge cut of *$G_h$*that meets the criteria of Lemma*2.1*, let*$R_1, R_2, \dots , R_l$ *form a partition of*$[vE(G_h)] .$ *Then,*$WL_{\Psi }(G_g, G_h)$ *is computed as the sum of *$EC_{\Psi }(R_j)$ *over all* $j$* from 1 to* $l$*, divided by* $v$.

##  Structure of $(K_{p}-C_{p})^{n}$

In the work^14^, on certain graphs and their Cartesian products, Sergie et al. used lexicographic order and a numerical characteristic value known as the $\delta -$sequence helps to achieve the optimality of the structure. The number of edges of the structure and its maximum subgraphs was considered an essential component of the research for embedding $(K_{p}-C_{p})^{n}$, but *p* is odd, $p > 9$, and $n \ge 2$, into suitable host graphs, which was considered a gap and worked by^9^. The symbol $\delta _{G}-$sequence represents the $\delta -$ sequence of a graph *G*. For a graph $G=(V,E)$ with *h* varying between 1 and the cardinality of its vertex set, the $\delta -$sequence is defined as the difference between the edges induced at *h* and the edges induced at $h-1$ index. The obvious condition that with a single vertex there are no edges that could be induced and hence $\delta (1)=0$.

The numeric character $\delta -$sequence of *G* is a sequence formed by the $\delta -$sequence of $1,2,\dots ,|V|$. The $delta-$sequence is said to be symmetric if for some $|V|=k$,

$$\delta (j)+ \delta (k-j+1)= \delta (p), \, \text {for} \, i = 1,\dots ,k$$

### Theorem 3.1

^14^*If lexicographic order is ideal for*$G \times G$*, then it is ideal for the *$n^{th}$* cartesian product of G for any *$n \ge 3$.

The structure $(K_p-C_p)$ is constructed using the $\delta$-sequence. Both the structure and its cartesian product are built through a recursive construction process. A method for determining the set that induce the maximum subgraph within $(K_p-C_p)^{n}$ for odd $p \ge 9$ and $n \ge 2$ is to set up the vertices of $(K_p-C_p)^{n}$ as in Theorem 3.1. According to^9^ in Theorem 3.3, the building of the structure is the strategy taken to solve the EIP for $(K_p-C_p)^{n}$.

### Lemma 3.1

^14^*For every odd *$p \ge 5$* the graph *$G_{p} = K_{p} - C_{p}$* has* $\delta$*-sequence,*

$$\delta _{G_{p}}(m) = {\left\{ \begin{array}{ll} m - 1 & \ 1 \le m \le (p-3)/2\\ \frac{(p-3)}{2} & \ (p-1)/2 \le m \le (p+3)/2\\ m-3 & \ (p+5) / 2 \le m \le p \end{array}\right. }$$

An significant conclusion from the lemma above is that $\delta _{G}$ is symmetric if and only if *G* is a graph with all elements in the set |*V*(*G*)| having the same degree. Because of the aforementioned, $(K_{p}-C_{p})^{n}$ is a regular graph, and its $\delta _{G}$ sequence is symmetric.

### Theorem 3.2

^14^ *For odd* $p \ge 9$ *or* $p = 5$ *, the lexicographic order for* $(K_{p}-C_{p})^{2}$ *is optimal.*

### Corollary 3.1

^14^*The optimal arrangement for *$$(K_{p}-C_{p})^{n},$$$$n \ge 2,$$ $$p \ge 9,$$* and p to be odd is the lexicographic order.*

The structure $(K_p - C_p)^{n}$ for odd $p \ge 9$, $n \ge 2$ in the first dimension has *p* vertices and each vertex is nonadjacent to two other vertices of the vertex set. The structure $(K_p - C_p)^{n-1}$ is repeated *p* times in $(K_p - C_p)^{n}$ construction with edges binding these *p* copies. For $n \ge 2$, $(K_p - C_p)^{n}$ structure has an underlying $p^{n-1} \times p^{n-1}$ grid. Each corresponding row and column of the underlying grid are isomorphic and induce subgraphs of $(K_p - C_p)^{n}$ that are maximum subgraphs. The Table 1 clearly states the parameters of the structure^9^ $(K_p - C_p)^{n}$ for odd $p \ge 9$, $n \ge 2$.

**Table 1:** Parameters of $(K_{p} - C_{p})^{n}$.

Parameters	$(K_{p} - C_{p})^{n}$
Node	$p^{n}$
Edge	$p^{n} \times \frac{(p-3)}{2}n$
Regularity	$(p-3)n$
Edge connectivity	$(p-3)n$
Girth	3

The structure $(K_{p}-C_{p})^{n}$ for $p=9$ was a novel structure studied in^15^, and its structural properties were analyzed, and further, the maximum subgraphs of the structure and an embedding function that mapped $(K_{9}-C_{9})^{n}$ as a guest graph to a few host graphs were determined, and the wirelength was computed in^15–18^ and the pancyclic property was the focus of study in^19^. The structure $(K_{p}-C_{p})^{n}$, for odd $p \ge 9$, $n \ge 2$, was further studied and identified the gap of edge isoperimetric problem in^9^ and embedded it in wheel-like graphs. This paper focuses on further exhausting graphs that could be embedded in $(K_{p}-C_{p})^{n}$, for odd $p \ge 9$, $n \ge 2$, and determining the parameter wirelength using the embedding function $\Psi$. The following theorems states the EIP of $(K_{p}-C_{p})^{n}$ by^9^ and the MinLA solved

### Theorem 3.3

^9^*If *$G_{g}$ *is* $(K_{p} - C_{p})^{n},$*p is odd, *$p \ge 9,$ $n \ge 2$*, for any integer h,* $0 \le h \le p^{n}$*, then*

$$\begin{aligned} & I_{(K_{p} - C_{p})^{n}}(h) = \left( p^{n-1} \times \frac{(p-3)}{2}(n-1)\right) x + \left( p^{n-1} \times I_{(K_{p} - C_{p})}(x)\right) +\left( I_{(K_{p} - C_{p})^{n-1}}(z)\right) + \left( z \times \delta (x+1)\right) \end{aligned}$$

*where *$x =\Big \lfloor \frac{h}{p^{n-1}} \Big \rfloor,$ $z = h \mod p^{n-1}$.* Also the maximum possible subgraph that can be formed with *$h$* vertices, along with the number of edges in that subgraph, is denoted by* $I_{G}(h)$.

### Theorem 3.4

^9^ *The minimum wirelength of embedding* $(K_{p}-C_{p})^{n}$ *into linear array* $L_{p^{n}}$ *, where p is odd,* $p > 9$ *and* $n \ge 2$ *is given by*

$$MinLA((K_{p}-C_{p})^{n}) = 2n(p-3) \left[ \frac{(p^{n}-1)(p^{n} - 3)}{4} \right] - 4 \sum _{j=1}^{\frac{p^{n}-1}{2}} I(j)$$

The scale of input has a direct correlation with time complexity. As the size of the input increases, so does the runtime, or the amount of time required for the algorithm to execute. As computers became increasingly common, computational efficiency emerged as a key concern. Time complexity, which quantifies the duration it takes a computer to execute an algorithm, became a key concept. Time complexity is one of the two primary sorts of computational difficulty in computer science. The other kind, space complexity, assesses how much memory an algorithm uses. The term “efficient” refers to algorithms that run in either linear or quadratic time. The MinLA problem, which asks how long it takes to embed the structure in a linear array in the shortest amount of time possible. The time complexity of Theorem 3.4 is achieved in linear time as stated below:

### MinLA algorithm of $(K_{p}-C_{p})^{n}$ and the time complexity

**Input: ** The graph $(K_{p}-C_{p})^{n}$ and linear array $L_{p^{n}}$ where *p* is odd, $p \ge 9$ and $n \ge 2$ with $p^{n}$ vertices.

**Algorithm: ** The host graph linear array $L_{p^{n}}$ is arranged and labeled as 1 from left of the array and continued in the right direction until $p^{n}-1$, and the graph $(K_{p}-C_{p})^{n}$ is labeled using lexicographic ordering.

**Output: ** To achieve the minimum wirelength and determine the algorithm’s time complexity is $O(X)$, where $X$ indicates linear time complexity, the expression $(K_{p} - C_{p})^{n}$ is embedded into the linear array $L_{p^{n}}$ using the function $\Psi$.

**Method: ** We use $X$ time units to label the vertices, where $(K_{p} - C_{p})^{n}$ consists of $X = p^{n}$ vertices. By applying the MinLA Algorithm, $p^{n} - 1$ convex edge cuts are generated, each requiring one time unit, resulting in $p^{n} - 1$ time units in total. Additionally, calculating $EC_{\Psi }$ on the linear array $L_{p^{n}}$ requires another $p^{n} - 1$ time units. Thus, computing the wirelength, as established by Theorem 3.4, requires $p^{n}$ time units. In total, the overall time required is $3p^{n} - 1$. Consequently, calculating an accurate MinLA of $(K_{p} - C_{p})^{n}$ has a linear time complexity of $O(X)$.

Further in this article, the main results on embedding $(K_{p}-C_{p})^{n}$ into certain graphs are discussed. An optimal embedding is alternatively referred to using terms such as best, desired, ideal, and preferable embedding. Also in this article an optimal wirelength refers to the minimum wirelength that is intended to be computed. In Sections “[Sec Sec5]”, “[Sec Sec7]”, “[Sec Sec9]”, “[Sec Sec11]” and “[Sec Sec13]”, the embedding of $(K_{p}-C_{p})^{n}$, where *p* is an odd number with $p \ge 9$, into grids, generalized book graphs, triangular snakes, extended banana trees, and arbitrarily fixed generalized banana trees is described using the function $\Psi$ and the solution of the edge isoperimetric problem. The optimal wirelengths of the respective embeddings are also obtained.

## Embedding $(K_{p}-C_{p})^{n}$ into $M(p^{\lfloor {\frac{n}{2}} \rfloor }, p^{\lceil {\frac{n}{2}} \rceil })$ grid

Optimizing the arrangement of elements is a crucial operation that speeds up data structure processing. Linear arrangement in data design and computing creates a linear sequence of structured data. It has a path like structure in graph theory. The cartesian product $H_{t_1} \times H_{t_2}$ is represented as $M(t_{1},t_{2})$ and is referred to as a grid where $H_{t_1}$ and $H_{t_2}$ are paths. $V(M(t_{1},t_{2})) = \{ v_{mn}: 1 \le m \le t_{1}, 1 \le n \le t_{2} \}$ is the definition of the grid’s vertex set and appropriately label the vertex as $v_{mn}$^20^.

### Theorem 4.1

*The wirelength* $WL_{\Psi }\left( (K_{p}-C_{p})^{n},M(p^{\lfloor {\frac{n}{2}} \rfloor }, p^{\lceil {\frac{n}{2}} \rceil }) \right)$ *for an optimal ordering gives the minimum wirelength of embedding* $(K_{p}-C_{p})^{n}$ *into* $M\left( p^{\lfloor {\frac{n}{2}} \rfloor }, p^{\lceil {\frac{n}{2}} \rceil }\right)$ *, where p is odd and* $p \ge 9$ *for* $n\ge 2$ *is given by*

1 $$\begin{aligned} & WL_{\Psi }\left( (K_{p}-C_{p})^{n},M(p^{\lfloor {\frac{n}{2}} \rfloor }, p^{\lceil {\frac{n}{2}} \rceil }) \right) = \nonumber \\ & \sum _{i=1}^{p^{\lceil \frac{n}{2} \rceil } - 1} [(p-3)n(i \times p^{\lfloor \frac{n}{2}\rfloor }) -2I(i \times p^{\lfloor \frac{n}{2}\rfloor })] + \sum _{j=1}^{p^{\lfloor \frac{n}{2} \rfloor } - 1} [(p-3)n(j \times p^{\lceil \frac{n}{2}\rceil }) -2I(j \times p^{\lceil \frac{n}{2}\rceil })]. \end{aligned}$$

### *Proof*

Let $(K_{p}-C_{p})^{n}$ and Grid, $M(p^{\lfloor {\frac{n}{2}} \rfloor }, p^{\lceil {\frac{n}{2}} \rceil })$ be $G_{(K_{p}-C_{p})^{n}}$ and $H_{M(p^{\lfloor {\frac{n}{2}} \rfloor }, p^{\lceil {\frac{n}{2}} \rceil })}$ respectively. Let $S^{'}_{i}$, $1 \le i \le p^{\lceil \frac{n}{2} \rceil } - 1$ and $T^{'}_{j}$, $1 \le j \le p^{\lfloor \frac{n}{2} \rfloor } - 1$ be the convex edge cuts which splits the grid into $H^{1}_{M(p^{\lfloor {\frac{n}{2}} \rfloor }, p^{\lceil {\frac{n}{2}} \rceil })}$ and $H^{2}_{M(p^{\lfloor {\frac{n}{2}} \rfloor }, p^{\lceil {\frac{n}{2}} \rceil })}$ whose inverse images are $G^{1}_{(K_{p}-C_{p})^{n}} = \Psi ^{-1}(H^{1}_{M(p^{\lfloor {\frac{n}{2}} \rfloor }, p^{\lceil {\frac{n}{2}} \rceil })})$ and $G^{2}_{(K_{p}-C_{p})^{n}} = \Psi ^{-1}(H^{2}_{M(p^{\lfloor {\frac{n}{2}} \rfloor }, p^{\lceil {\frac{n}{2}} \rceil })})$ respectively. The components of the edge cut $S^{'}_{i}$, have $|V(H^{1})_{M(p^{\lfloor {\frac{n}{2}} \rfloor }, p^{\lceil {\frac{n}{2}} \rceil })} | = p^{\lfloor \frac{n}{2} \rfloor } i$ and $|V(H^{2})_{M(p^{\lfloor {\frac{n}{2}} \rfloor }, p^{\lceil {\frac{n}{2}} \rceil })} | = (p^{\lceil \frac{n}{2}\rceil } - i) p^{\lfloor \frac{n}{2}\rfloor }$ and the components by the edge cut $T^{'}_{j}$, have $|V(H^{1})_{M(p^{\lfloor {\frac{n}{2}} \rfloor }, p^{\lceil {\frac{n}{2}} \rceil })} | = p^{\lceil \frac{n}{2} \rceil } j$ and $|V(H^{2})_{M(p^{\lfloor {\frac{n}{2}} \rfloor }, p^{\lceil {\frac{n}{2}} \rceil })} | = (p^{\lfloor \frac{n}{2}\rfloor } - j) p^{\lceil \frac{n}{2}\rceil }$. The convex edge cuts $S^{'}_{i}$ and $T^{'}_{j}$ satisfy the conditions of Lemma 2.1, thus $EC_{\Psi }(S^{'}_{i})$ and $EC_{\Psi }(T^{'}_{j})$ be the minimum edge congestion of the embedding $\Psi$. From Lemma 2.1 we have the minimum edge congestion values, for $1 \le i \le p^{\lceil \frac{n}{2} \rceil } - 1$, $EC_{\Psi }(S^{'}_{i})$ and for $1 \le j \le p^{\lfloor \frac{n}{2} \rfloor } -1$, $EC_{\Psi }(T^{'}_{j})$ are given as,

2 $$\begin{aligned} EC_{\Psi }(S^{'}_{i})= & (p-3)n(i \times p^{\lfloor \frac{n}{2}\rfloor }) -2I(i \times p^{\lfloor \frac{n}{2}\rfloor }) \end{aligned}$$

3 $$\begin{aligned} EC_{\Psi }(T^{'}_{j})= & (p-3)n(j \times p^{\lceil \frac{n}{2}\rceil }) -2I(j \times p^{\lceil \frac{n}{2}\rceil }) \end{aligned}$$

Thus Eqs. 2, 3 and Lemma 2.2 results in the minimum wirelength for embedding $(K_{p}-C_{p})^{n}$ into $M\left( p^{\lfloor {\frac{n}{2}} \rfloor }, p^{\lceil {\frac{n}{2}} \rceil }\right)$, where *p* is odd and $p \ge 9$ for $n\ge 2$, is given by

$$\begin{aligned} WL_{\Psi }\left( (K_{p}-C_{p})^{n},M(p^{\lfloor {\frac{n}{2}} \rfloor }, p^{\lceil {\frac{n}{2}} \rceil }) \right) = \sum _{i=1}^{p^{\lceil \frac{n}{2} \rceil } - 1}EC_{\Psi }(S^{'}_{i}) + \sum _{j=1}^{p^{\lfloor \frac{n}{2} \rfloor } - 1}EC_{\Psi }(T^{'}_{j}). \end{aligned}$$

Consequently, the wirelength value from the above equation is as minimal as in Eq. 1. $\square$

**Algorithm 1:**
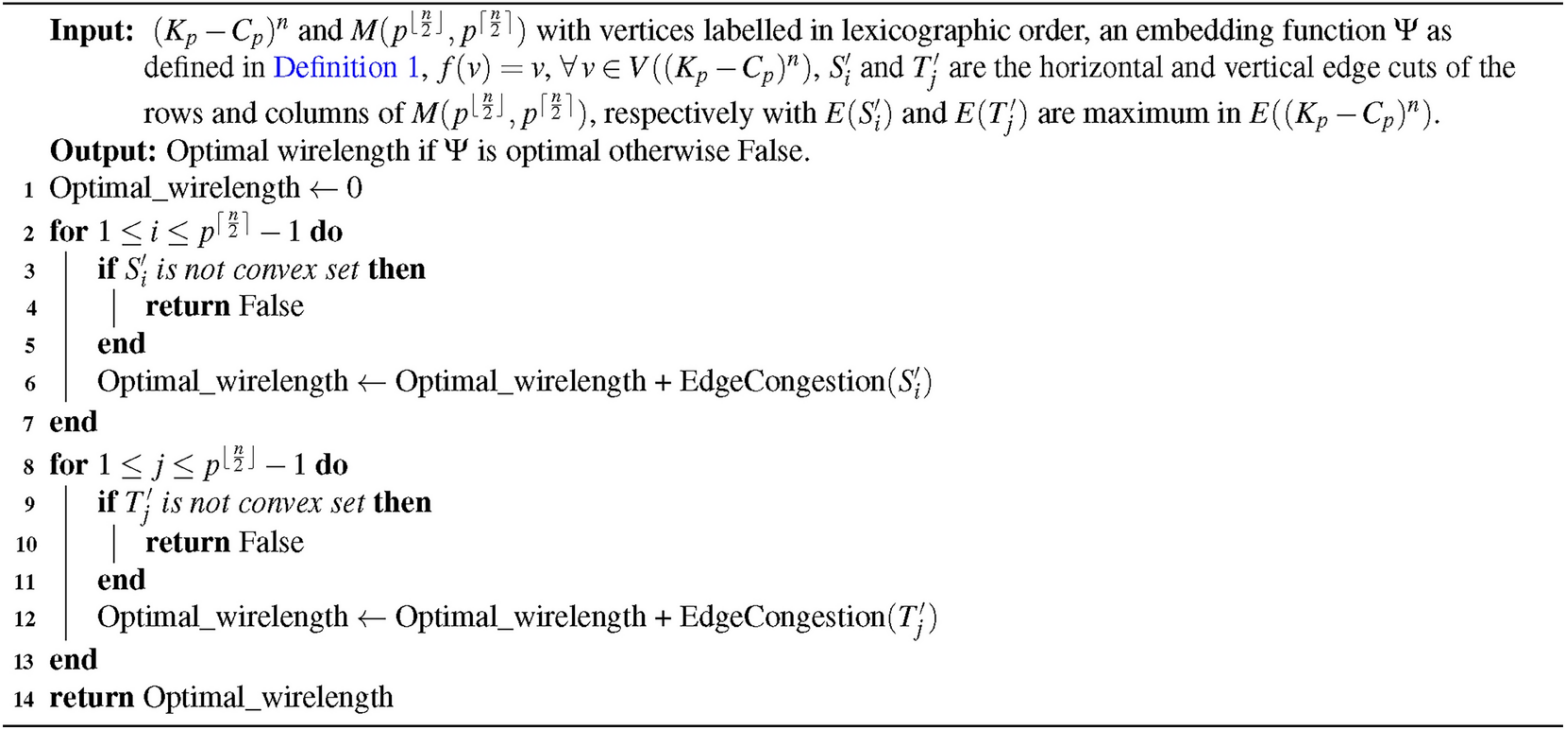
Optimal wirelength for embedding $(K_{p}-C_{p})^{n}$ into $M(p^{\lfloor {\frac{n}{2}} \rfloor }, p^{\lceil {\frac{n}{2}} \rceil })$

### Time complexity of the algorithm

**Input: ** The structure $(K_{p} - C_{p})^{n}$ and $M(p^{\lfloor \frac{n}{2} \rfloor }, p^{\lceil \frac{n}{2} \rceil })$, where $p$ is an odd number with $p \ge 9$, and $n \ge 1$, both consist of $p^{n}$ vertices.

**Algorithm: ** The embedding process described in algorithm 1.

**Output: ** The duration required to execute embedding algorithm 1.

**Method: ** Let $X=p^{n}$ be the be the number of vertices. Then the time complexity of the above algorithm 1 is,

$$\begin{aligned}&=p^{n} + 2p^{\lceil {\frac{n}{2}}\rceil } + 2p^{\lfloor {\frac{n}{2}}\rfloor }-3 \\&\le p^{n} \end{aligned}$$

Thus, calculating the minimum wirelength for embedding $(K_{p} - C_{p})^{n}$ into the grid requires $O(X)$, which corresponds to linear time complexity.

## Embedding $(K_{p}-C_{p})^{n}$ into $GB(p^{\left\lfloor \frac{n-1}{2}\right\rfloor },p^{\lceil \frac{n-1}{2}\rceil }, p)$ generalized book graph

Consider a $t_1 \times t_2$ mesh *M* with $t_1$ rows and $t_2$ columns. By connecting each vertex of the $1^{st}$ column of $M_1$ to the corresponding vertex of the $1^{st}$ column of $M_i$ via an edge for all $i = 2,3, \dots , l$, a graph that is created from *l* copies of *M*, say $M_1, M_2, \dots , M_l$, is known as a generalized book and is represented by $GB[t_1,t_2,l]$. For $GB[t_{1},t_{2},l]$ there are $t_{1}t_{2}l$ vertices and $t_{2}l(2t_{1}-1)-t_{1}$ edges around $t_{1}+2t_{2}-1$ is the diameter $d(GB[t_{1},t_{2},l])$.

### Theorem 5.1

*The minimum wirelength of embedding *$$(K_{p}-C_{p})^{n}$$* into Generalized Book Graph* $$GB(p^{\left\lfloor \frac{n-1}{2}\right\rfloor },p^{\big \lceil \frac{n-1}{2}\big \rceil }, p )$$*, for p is odd, *$$p \ge, 9$$$$n \ge 3$$* is given by,*

4 $$\begin{aligned} & WL_{\Psi }\left( (K_{p}-C_{p})^{n},GB(p^{\left\lfloor \frac{n-1}{2}\right\rfloor },p^{\big \lceil \frac{n-1}{2}\big \rceil }, p)\right) = \sum _{i=1}^{p^{\left\lfloor \frac{n-1}{2}\right\rfloor }-1} \left[ (p-3)np^{\big \lceil \frac{n-1}{2} \big \rceil }pi -2I(p^{\big \lceil \frac{n-1}{2} \big \rceil }pi) \right] + \nonumber \\ & \sum _{j=1}^{\left( p^{\big \lceil \frac{n-1}{2}\big \rceil }-1 \right) p} \left[ (p-3)n p^{\left\lfloor \frac{n-1}{2}\right\rfloor }(j+(\alpha -1)) - 2I\left( p^{\left\lfloor \frac{n-1}{2}\right\rfloor }(j+(\alpha -1))\right) \right] + \nonumber \\ & \sum _{h=1}^{p-1} \left[ (p-3)nhp^{n-1} - 2I(hp^{n-1})\right] \text {where} \quad \alpha = \big \lceil \frac{j}{p^{\lceil \frac{n-1}{2}\rceil } -1}\big \rceil \end{aligned}$$

### *Proof*

Let $(K_{p}-C_{p})^{n}$ and Generalized Book Graph $GB(p^{\left\lfloor \frac{n-1}{2}\right\rfloor },p^{\big \lceil \frac{n-1}{2}\big \rceil }, p )$, for *p* is odd, $p \ge 9$, $n \ge 3$ be represented as $G_{(K_{p}-C_{p})^{n}}$ and $H_{GB(p^{\left\lfloor \frac{n-1}{2}\right\rfloor },p^{\big \lceil \frac{n-1}{2}\big \rceil }, p )}$ respectively. By Theorem 3.1 and its Corollary 3.1 the optimal ordering is given for the vertices of $(K_{p}-C_{p})^{n}$. Thus the optimal ordering for the vertices of $G_{(K_{p}-C_{p})^{n}}$ and $H_{GB(p^{\left\lfloor \frac{n-1}{2}\right\rfloor },p^{\big \lceil \frac{n-1}{2}\big \rceil }, p )}$ is as in Algorithm 2. The sets $S_{i}$, where $1 \le i \le p^{\lfloor \frac{n-1}{2} \rfloor } - 1$, $T_{j}$, where $1 \le j \le (p^{\lceil \frac{n-1}{2} \rceil } - 1)p$, and $K_{h}$, where $1 \le h \le p-1$, represent the convex edge cuts of the graph $H_{GB(p^{\lfloor \frac{n-1}{2} \rfloor }, p^{\lceil \frac{n-1}{2} \rceil }, p)}$. These cuts partition the graph into two components, denoted as $H^1_{GB(p^{\lfloor \frac{n-1}{2} \rfloor }, p^{\lceil \frac{n-1}{2} \rceil }, p)}$ and $H^2_{GB(p^{\lfloor \frac{n-1}{2} \rfloor }, p^{\lceil \frac{n-1}{2} \rceil }, p)}$. For the embedding function $\Psi$, the components $H^{1}_{GB(p^{\left\lfloor \frac{n-1}{2}\right\rfloor },p^{\big \lceil \frac{n-1}{2}\big \rceil }, p )}$ and $H^{2}_{GB(p^{\left\lfloor \frac{n-1}{2}\right\rfloor },p^{\big \lceil \frac{n-1}{2}\big \rceil }, p )}$ correspond to the preimages of $G^{1}_{(K_{p}-C_{p})^{n}}$ and $G^{2}_{(K_{p}-C_{p})^{n}}$, respectively. Consequently, the convex edge cuts determine the optimal subgraph in $(K_{p}-C_{p})^{n}$, as stated in Theorem 3.3.

The conditions of Lemma 2.1 are satisfied by the convex edge cuts $S_{i}$, $T_{j}$, and $K_{h}$. Their respective minimum edge congestions are denoted by $EC_{\Psi }(S_{i})$, $EC_{\Psi }(T_{j})$, and $EC_{\Psi }(K_{h})$. According to Lemma 2.1, the minimum edge congestion for $1 \le i \le p^{\lfloor \frac{n-1}{2}\rfloor } - 1$ is $EC_{\Psi }(S_{i})$, for $1 \le j \le (p^{\lceil \frac{n-1}{2}\rceil } - 1)p$ is $EC_{\Psi }(T_{j})$, and for $1 \le h \le p - 1$ is $EC_{\Psi }(K_{h})$, given by:

5 $$\begin{aligned} EC_{\Psi }(S_{i})= & (p-3)npip^{\lceil \frac{n-1}{2}\rceil } -2I(pip^{\lceil \frac{n-1}{2}\rceil }) \end{aligned}$$

6 $$\begin{aligned} EC_{\Psi }(T_{j})= & (p-3)n(j+(\alpha - 1)) p^{\lfloor \frac{n-1}{2}\rfloor } - 2I((j+(\alpha - 1)) p^{\lfloor \frac{n-1}{2}\rfloor }) \quad \text {where} \quad \alpha = \lceil \frac{j}{p^{\lceil \frac{n-1}{2}\rceil } -1}\rceil \end{aligned}$$

7 $$\begin{aligned} EC_{\Psi }(K_{h})= & (p-3)nhp^{n-1} - 2I(hp^{n-1}) \end{aligned}$$

According to Lemma 2.2, the minimum desirable wirelength of the embedding function $\Psi$ is determined using Eqs. 5, 6, and 7 as follows:

$$\begin{aligned} WL_{\Psi }\left( (K_{p}-C_{p})^{n}, GB(p^{\left\lfloor \frac{n-1}{2}\right\rfloor },p^{\big \lceil \frac{n-1}{2}\big \rceil }, p )\right)&= \sum _{i=1}^{p^{\left\lfloor \frac{n-1}{2}\right\rfloor }-1} EC_{\Psi }(S_{i}) + \sum _{j=1}^{\left( p^{\big \lceil \frac{n-1}{2}\big \rceil }-1 \right) p} EC_{\Psi }(T_{j}) + \sum _{h=1}^{p-1} EC_{\Psi }(K_{h}) \end{aligned}$$

Hence, the minimum wirelength for embedding $(K_{p} - C_{p})^{n}$ into $GB(p^{\left\lfloor \frac{n-1}{2}\right\rfloor }, p^{\big \lceil \frac{n-1}{2}\big \rceil }, p)$, where *p* is odd, $p \ge 9$, and $n \ge 3$, using the embedding function $\Psi$, is provided as in Eq. 4. $\square$

**Algorithm 2:**
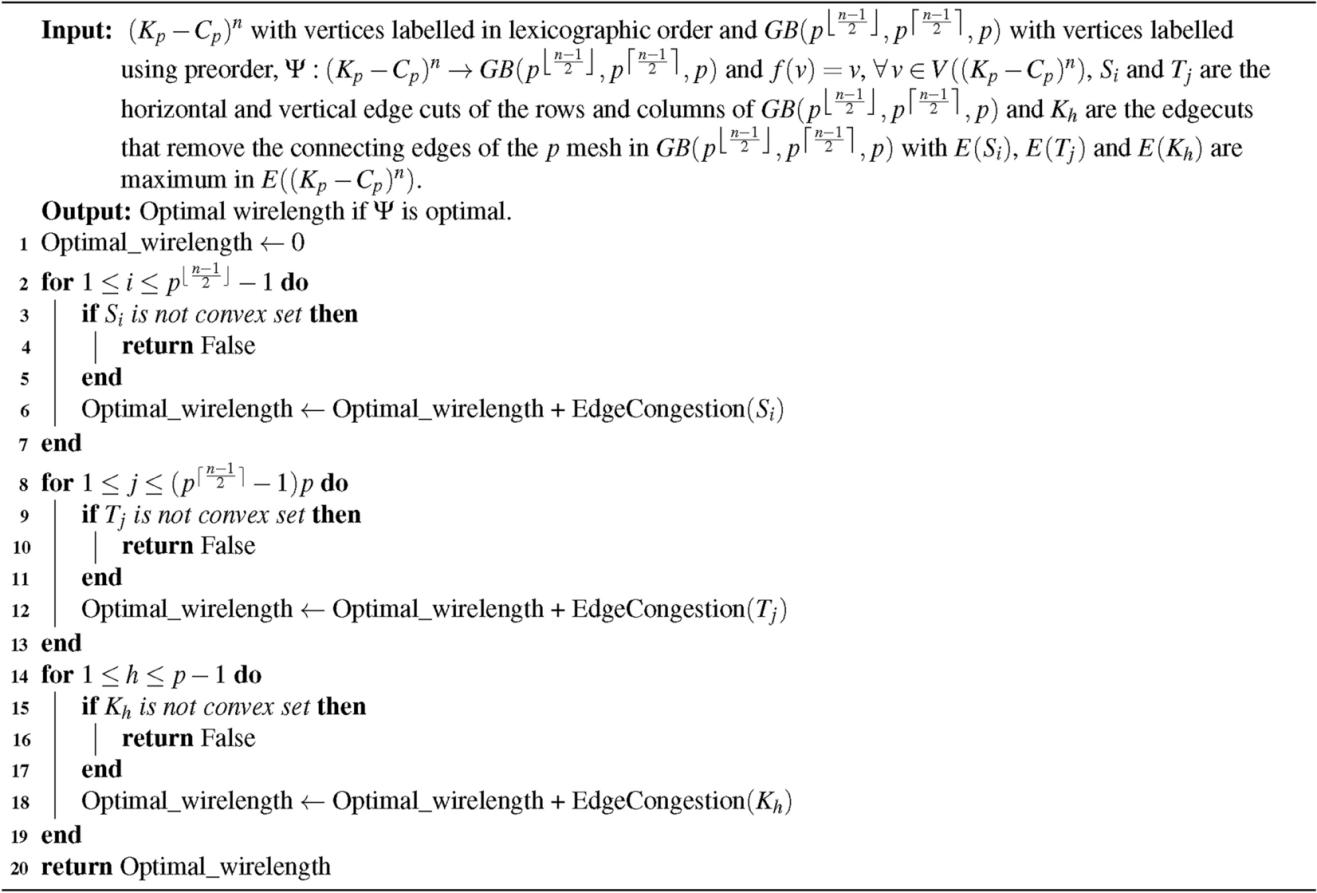
Optimal wirelength for embedding $(K_{p}-C_{p})^{n}$ into $GB(p^{\left\lfloor \frac{n-1}{2}\right\rfloor },p^{\big \lceil \frac{n-1}{2}\big \rceil }, p)$

### Time complexity of the algorithm

**Input:** $(K_{p}-C_{p})^{n}$ and Generalized Book Graph $GB(p^{\left\lfloor \frac{n-1}{2}\right\rfloor },p^{\big \lceil \frac{n-1}{2}\big \rceil }, p )$ where *p* is odd, $p \ge 9$ and $n \ge 3$ with $p^{n}$ vertices.

**Algorithm: ** The embedding algorithm 2.

**Output: ** The duration required to execute embedding algorithm 2.

**Method: ** The time complexity of the algorithm depends on optimally ordering the vertices, consider as *X*, identifying the convex edge cuts, computing $EC_{\Psi }$ and computing the minimum $WL_{\Psi }$ for the embedding $\Psi$ is given by,

$$\begin{aligned}&= p^{\lfloor \frac{n-1}{2}\rfloor } + pp^{\lceil \frac{n-1}{2}\rceil } - 2\\&\le p^{n} \end{aligned}$$

Therefore, the time required to compute the minimum wirelength for embedding $(K_{p}-C_{p})^{n}$ into $GB(p^{\left\lfloor \frac{n-1}{2}\right\rfloor }, p^{\big \lceil \frac{n-1}{2}\big \rceil }, p)$ is $O(X)$, which corresponds to linear time complexity.

## Embedding $(K_{p}-C_{p})^{n}$ into $TS_{p^n}$ triangular snake

A triangular snake $TS_l$ with $l$ vertices is constructed from the path $v_1, v_2, \dots , v_{\lfloor \frac{l}{2} \rfloor + 1}$ by adding a vertex $u_t$ between each pair of consecutive vertices $v_t$ and $v_{t+1}$, for $t = 1, 2, \dots , \lceil \frac{l}{2} \rceil - 1$^21^.

### Theorem 6.1

*The minimum wirelength of embedding* $(K_{p}-C_{p})^{n}$ *into* $TS_{p^n}$ *, for p is odd,* $p \ge 9,$ $n \ge 1$ *is given by*

8 $$\begin{aligned} WL_{\Psi }((K_{p}-C_{p})^{n},TS_{p^n}) = \frac{n(p-3)(p^{n}-1)}{4} + \frac{1}{2}\sum _{j=1}^{ 2 \left( \big \lfloor \frac{p^{n}}{2} \big \rfloor -1 \right) + 2} \big ((p-3)nj - 2 I(j)\big ). \end{aligned}$$

### *Proof*

Let $(K_{p}-C_{p})^{n}$ and Triangular Snake $TS_{p^{n}}$ be $G_{(K_{p}-C_{p})^{n}}$ and $H_{TS_{p^{n}}}$ respectively. The ordering that induce optimal subgraph in $(K_{p}-C_{p})^{n}$ is obtained by using the Theorem 3.1 and its Corollary 3.1. Thus, the vertices of the triangular snake $TS_{p^{n}}$ and the structure $(K_{p}-C_{p})^{n}$ are labeled using preorder traversal and lexicographic ordering, respectively. The convex edge cuts of $TS_{p^{n}}$ are $S_{i}$, $1 \le i \le \left\lfloor \frac{p^{n}}{2} \right\rfloor$ and $T_{j}$, $1 \le j \le 2 \left( \big \lfloor \frac{p^{n}}{2} \big \rfloor -1 \right) + 2$. These convex edge cuts split $TS_{p^{n}}$ into exactly two components. Let $H^{1}_{TS_{p^{n}}}$ and $H^{2}_{TS_{p^{n}}}$ be the first and the second component whose inverse images are $G^{1}_{(K_{p}-C_{p})^{n}} = \Psi ^{-1}(H^{1}_{TS_{p^{n}}})$ and $G^{2}_{(K_{p}-C_{p})^{n}} = \Psi ^{-1}(H^{2}_{TS_{p^{n}}})$ respectively. The cardinality of the vertex set of the two components of $TS_{p^{n}}$ by the convex cut $S_{i}$ is $|V(H^{1}_{TS_{p^{n}}})|= 1$ and $|V(H^{2}_{TS_{p^{n}}})| =(p^{n}-1)$ respectively. In addition, $|V(H^{1}_{TS_{p^{n}}})|=j$, is the cardinality of the first component and $|V(H^{2}_{TS_{p^{n}}})| = (p^{n} - j)$, is the cardinality of the second component, for $T_{j}$, the convex edge cut. According to Theorem 3.3, the optimal subgraph in $(K_{p}-C_{p})^{n}$ is thus induced by the convex edge cuts.

As a result, the partitions $S_{i}$ and $T_{j}$ satisfy the objectives in Lemma 2.1, and their respective minimum edge congestion is $EC_{\Psi }(S_{i})$ and $EC_{\Psi }(T_{j})$.

According to Lemma 2.1, the optimum values of edge congestion for $1 \le i \le \left\lfloor \frac{p^{n}}{2} \right\rfloor$, $EC_{\Psi }(S_{i})$ and for $1 \le j \le 2 \left( \big \lfloor \frac{p^{n}}{2} \big \rfloor -1 \right) + 2$, $EC_{\Psi }(T_{j})$ are given as,

9 $$\begin{aligned} EC_{\Psi }(S_{i})= & (p-3)n \end{aligned}$$

10 $$\begin{aligned} EC_{\Psi }(T_{j})= & (p-3)nj - 2 I(j) \end{aligned}$$

For *p* being odd, $p \ge 9$, and $n \ge 1$, the minimal wirelength of embedding $(K_{p}-C_{p})^{n}$ into $TS_{p^n}$ is determined by Lemma 2.2 and Eqs. 9 and 10.

$$\begin{aligned} WL_{\Psi }((K_{p}-C_{p})^{n},TS_{p^n}) = \frac{1}{2}\sum _{i = 1}^{\left\lfloor \frac{p^{n}}{2} \right\rfloor } EC_{\Psi }(S_{i}) + \frac{1}{2}\sum _{j = 1}^{ 2 \left( \left\lfloor \frac{p^{n}}{2} \right\rfloor -1 \right) + 2} EC_{\Psi }(T_{j}). \end{aligned}$$

Thus the minimum wirelength of embedding $\Psi : (K_{p}-C_{p})^{n} \rightarrow TS_{p^{n}}$, is proved as given in Eq. 8. $\square$

**Algorithm 3:**
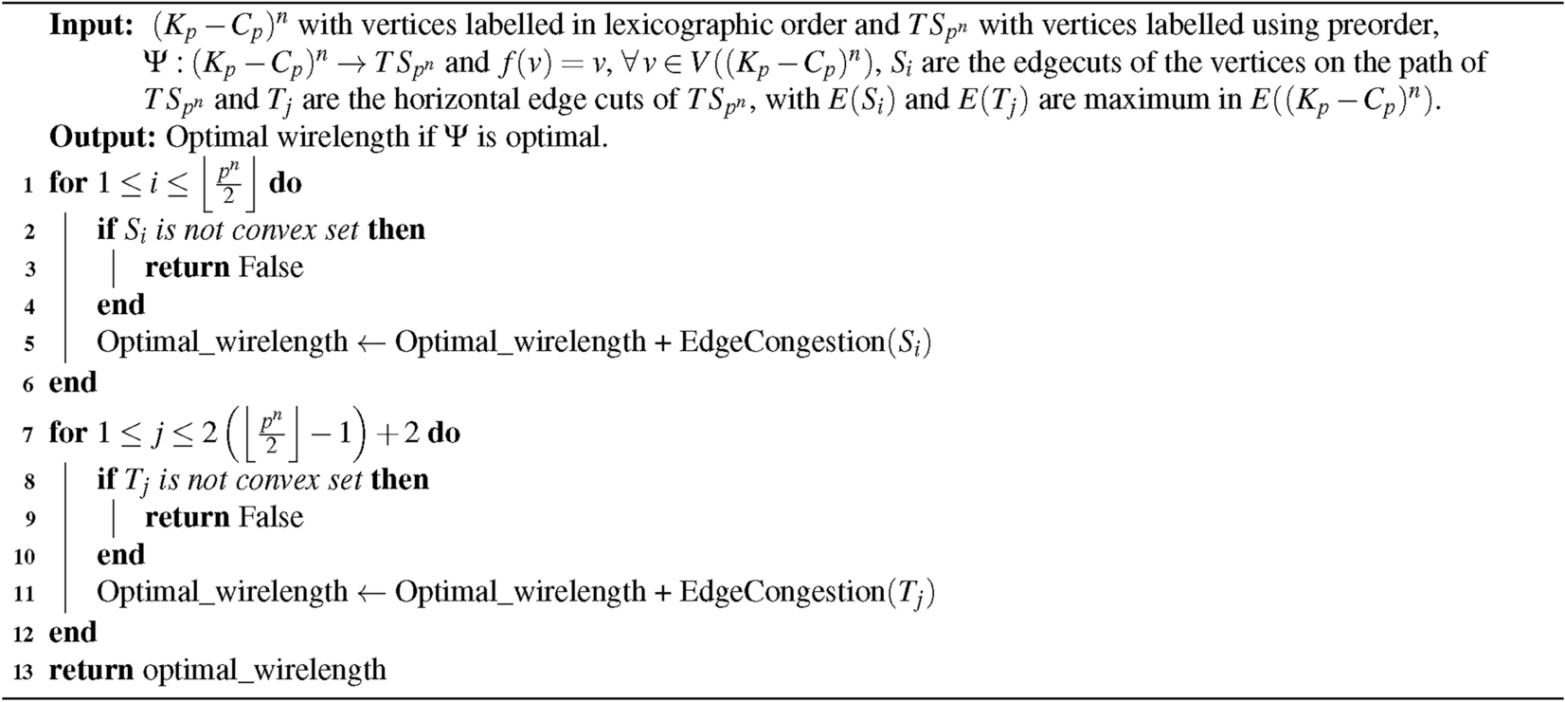
Optimal wirelength for embedding $(K_{p}-C_{p})^{n}$ into $TS_{p^{n}}$

### Time complexity of the algorithm

**Input:** $(K_{p}-C_{p})^{n}$ and Triangular Snake $TS_{p^{n}}$ where *p* is odd, $p \ge 9$ and $n \ge 1$ with $p^{n}$ vertices.

**Algorithm: ** The procedure is described in embedding algorithm 3.

**Output: ** The time required to compute and execute algorithm 3 for the embedding $\Psi : (K_p - C_p)^n \rightarrow TS_{p^n}$.

**Method: ** Let $X = p^n$ represent the number of vertices. Labeling these $X$ vertices requires $X$ time units. Using algorithm 3, it is expected to generate $\frac{3}{2}(p^n - 1)$ convex edge cuts, which will take $\frac{3}{2}(p^n - 1)$ time units, as each edge cut requires one unit of time. Additionally, calculating the edge congestion for these convex edge cuts will consume another $\frac{3}{2}(p^n - 1)$ time units. Finally, one additional time unit is required to compute the wirelength as stated in Theorem 6.1. Hence, the total time required can be expressed as follows:

$$\begin{aligned}&=p^{n} + \frac{3}{2}(p^{n} - 1) +\frac{3}{2}(p^{n} - 1) + 1 \\&= 2(2p^{n} - 1) \\&\le 4X - 2 \end{aligned}$$

A linear time *O*(*X*) is thus required to calculate the minimal wirelength for embedding of $(K_{p}-C_{p})^{n}$ into $TS_{p^{n}}$.

## Embedding $(K_{p}-C_{p})^{n}$ into $B(2, 1; k_1, k_2)$ extended banana tree

A Banana Tree, $B(r, s)$, is formed by attaching one leaf from each of the $r$ copies of an $s$-star graph to a single root vertex that is not part of any of the star graphs.

An Extended Banana Tree, represented as $B(r_1, r_2, \dots , r_m; s_1, s_2, \dots , s_m)$, is constructed by connecting one leaf from each of the $r_1, r_2, \dots , r_m$ copies of the $s_1, s_2, \dots , s_m$-star graphs to a vertex which is chosen to be the root, which is distinct from the vertices of the star graphs.

### Theorem 7.1

*The optimal wirelength of embedding *$(K_{p}-C_{p})^{n}$* into Extended Banana Tree *$B(n_t; k_t)$*, for p is odd, *$p \ge 9,$ $n \ge 2$* and *$1 \le t \le t_{0},$*where *$t_{0} \ge 2$*, is given by,*

11 $$\begin{aligned} WL_{\Psi }\left( (K_{p}-C_{p})^{n}, B(n_t; k_t)\right) = \sum _{i=1}^{t_{0}} \Big ( ( (p-3)n k_{i} ) - 2I(k_{i}) \Big ) + (p-3)n \Big ( \sum _{h=1}^{t_{0}}k_{h}-t_{0} \Big ) \end{aligned}$$

### *Proof*

Let $(K_{p}-C_{p})^{n}$ and Extended Banana Tree $B(n_t; k_t)$, for *p* is odd, $p \ge 9$, $n \ge 2$ and $1 \le t \le t_{0}$ be represented as $G_{(K_{p}-C_{p})^{n}}$ and $H_{B(n_t; k_t)}$ respectively. The vertices of $G_{(K_{p}-C_{p})^{n}}$ are arranged optimally using Theorem 3.1 and Corollary 3.1. The vertices of $H_{B(n_t; k_t)}$, where $1 \le t \le t_{0}$ is labeled using the preorder traversal which is its optimal ordering. The single root vertex is connected to one pendant vertex of each of the, $t_{0} = 3$ star graphs, each of which has $k_1, k_2, k_3$ vertices, to create the Extended Banana tree $H_{B(n_t; k_t)}$. Let $S_{i}$, $1 \le i \le t_{0}$ and $S^{'}_{j}$, $1 \le j \le p^{n}-(t_{0}+1)$ be convex cuts that disconnects $H_{B(n_{t},k_{t})}$ into two components. Thus the components $H_{B(n_t; k_t)}^{1}$ and $H_{B(n_t; k_t)}^{2}$ are the optimal subgraphs satisfying Theorem 3.3 which are the inverse images of $G_{(K_p - C_p)^{n}}^{1}$ and $G_{(K_p - C_p)^{n}}^{2}$ for the embedding function $\Psi$. The convex edge cuts $S_{i}$ and $S_{j}^{'}$ satisfy Lemma 2.1 which results in the minimum edge congestion $EC_{\Psi }(S_{i})$ for $1 \le i \le t_{0}$ and $EC_{\Psi }(S_{j}^{'})$ for $1 \le j \le p^{n}-(t_{0}+1)$ that is given as,

12 $$\begin{aligned} EC_{\Psi }(S_{i})= & \Big ((p-3)n k_{i} \Big ) - 2I(k_{i}) \end{aligned}$$

13 $$\begin{aligned} EC_{\Psi }(S_{j}^{'})= & (p-3)n \end{aligned}$$

Thus by Lemma 2.2 the minimum wirelength for the embedding function $\Psi : G_{(K_{p}-C_{p})^{n}} \rightarrow H_{B(n_t; k_t)}$ using Eqs. 12 and 13 is given as follows,

14 $$\begin{aligned} WL\left( (K_{p}-C_{p})^{n}, B(n_t; k_t)\right)&= \sum _{i=1}^{t_{0}}EC_{\Psi }(S_{i}) + \sum _{j=1}^{p^{n}-(t_{0}+1)}EC_{\Psi }(S_{j}^{'}) \nonumber \\&= \sum _{i=1}^{t_{0}} \Big ( ( (p-3)n k_{i} ) - 2I(k_{i}) \Big ) + (p-3)n \Big ( \sum _{i=1}^{t_{0}}k_{i}-t_{0} \Big ) \end{aligned}$$

**Fig. 1:**
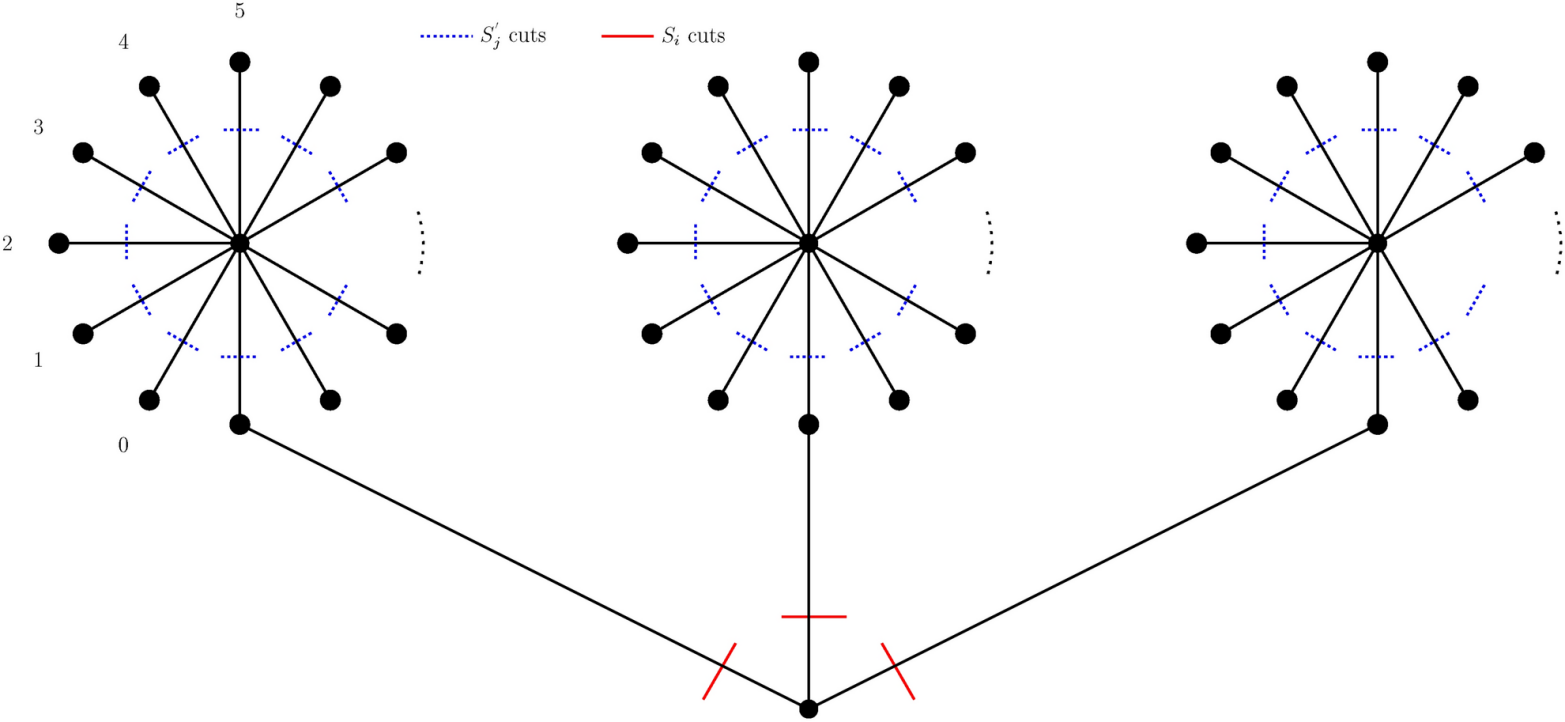
Extended Banana Tree $B(n_1,n_2,n_3; k_1,k_2,k_3)$ with each star graph consisting of $k_1$, $k_2$ and $k_3$ vertices respectively.

A general representation of the extended banana tree is given in Fig. 1. The above equation is further calculated for $B(n_t; k_t)$, $1 \le t \le t_{0}$ as cases depending on *p* and *n* as the following:

**Case 1:** when $$p \equiv 0 \mod 3$$

The graph $H_{B(n_1,n_2,n_3; k_1,k_2,k_3)}$, connects a root vertex to star graphs each with $n_1=n_2=n_3=1$, $k_{1}=k_{2}=\big \lceil \frac{p^{n}-1}{3} \big \rceil$ and $k_{3}=\left\lfloor \frac{p^{n}-1}{3} \right\rfloor$ vertices respectively. For convinence let us represent the Extended Banana Tree for this particular case as $B(2,1;\lceil \frac{p^{n}-1}{3} \rceil ,\lfloor \frac{p^{n}-1}{3} \rfloor )$ whose wirelength follows from Eq. 14 for the respective values.

**Case 2:** when $$p \equiv 1 \mod 3$$

The graph $H_{B(n_1,n_2,n_3; k_1,k_2,k_3)}$ connects a root vertex to star graphs each with $n_1=n_2=n_3=1$, $k_{1}=k_{2}=k_{3}=\frac{p^{n}-1}{3}$ vertices. Let us represent the Extended Banana Tree as $B(3;\frac{p^{n}-1}{3} )$ whose wirelength follows from Eq. 14 for the respective values.

**Case 3a:** when $p \equiv 2 \mod 3$ and *n* is even

This particular case follows as in Case 2 and thus the wirelength follows.

**Case 3b:** when $p \equiv 2 \mod 3$ and *n* is odd

The graph $H_{B(n_1,n_2,n_3; k_1,k_2,k_3)}$, connects a root vertex to three star graphs with $n_1=n_2=n_3=1$, $k_{1}=k_{2}=\left\lfloor \frac{p^{n}-1}{3} \right\rfloor$ and $k_{3}=\big \lceil \frac{p^{n}-1}{3} \big \rceil$ vertices respectively. Let us represent the Extended Banana Tree for this particular case as $B(2,1;\lfloor \frac{p^{n}-1}{3} \rfloor , \lceil \frac{p^{n}-1}{3} \rceil )$ whose wirelength follows from Eq. 14 for the respective values. $\square$

**Algorithm 4:**
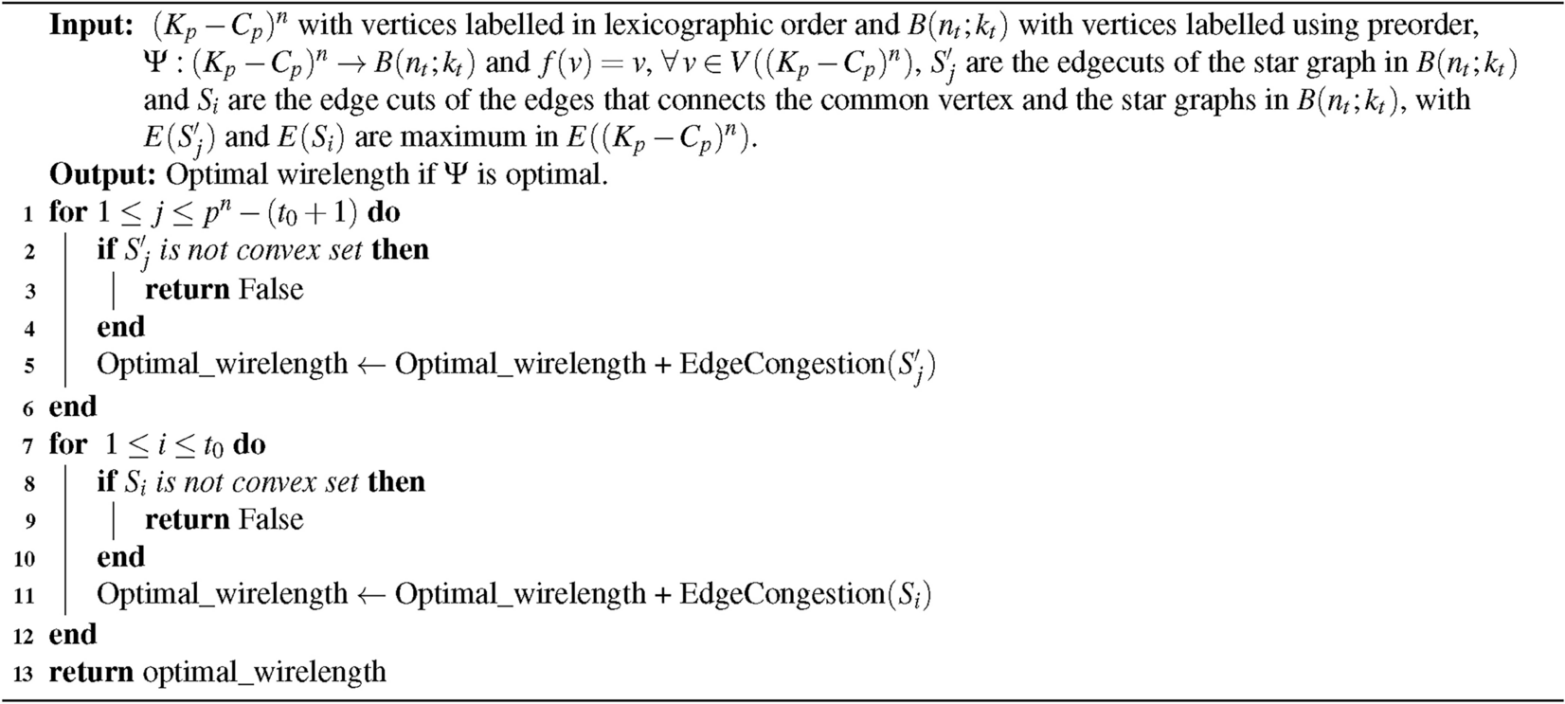
Optimal wirelength for embedding $(K_{p}-C_{p})^{n}$ into $B(n_t; k_t)$

###  Time complexity of the algorithm

**Input:** $(K_{p}-C_{p})^{n}$ and Extended Banana Tree $B(n_t; k_t)$ where *p* is odd, $p \ge 9$ and $n \ge 1$ with $p^{n}$ vertices.

**Algorithm: ** The embedding algorithm 4.

**Output: ** The time needed for the execution of the embedding algorithm 4.

**Method: ** The time complexity of the algorithm depends on optimally ordering the vertices, consider as *X*, identifying the convex edge cuts, computing the minimum edge congestion and computing the minimum wirelength of the embedding $\Psi$ which is given by,

$$\begin{aligned}&= 1+ p^{n} + 3 + p^{n}-4 \\&\le p^{n} \end{aligned}$$

Therefore, using the function $\Psi$ to get the ideal wirelength for embedding $(K_{p}-C_{p})^{n}$ into Extended Banana Tree $B(n_t; k_t)$ takes *O*(*X*), which is linear.

## Embedding $(K_{p}-C_{p})^{n}$ into $T(2, \left\lfloor \frac{p^{n} - 1}{4} \right\rfloor , k)$ arbitrarily fixed generalized banana tree

The rooted Arbitrarily Fixed Generalized Banana Tree with *l* copies of *C*(*g*, *h*), where *C*(*g*, *h*) represents a caterpillar and is denoted by *T*(*l*, *g*, *h*). A caterpillar is a connected acyclic graph in which the spine, or backbone, is produced by eliminating the legs, or vertices of degree one. Let *g* and *h* correspond to the spine and legs respective numbers of vertices.

### Theorem 8.1

*The minimum wirelength of embedding* $(K_{p}-C_{p})^{n}$* into Arbitrarily Fixed Generalized Banana Tree *$T(2, \left\lfloor \frac{p^{n} - 1}{4} \right\rfloor , k)$*, for p is odd, *$p \ge 9,$ $n \ge 1$*, is given in the following theorem*.

### *Proof*

Let $\Psi$ be an embedding function that embeds $(K_p -C_p)^{n}$ into $T(2, \left\lfloor \frac{p^{n} - 1}{4} \right\rfloor , k)$, for *p* is odd, $p \ge 9$, $n \ge 1$, consider the structures as $G_{(K_p -C_p)^{n}}$ and $H_{T(2, \left\lfloor \frac{p^{n} - 1}{4} \right\rfloor , k)}$ respectively. The preordering and Theorem 3.1 are used to label the vertices of $T(2, \left\lfloor \frac{p^{n} - 1}{4} \right\rfloor , k)$ and $(K_p -C_p)^{n}$, respectively. The maximum subgraph is identified for the optimal ordering as in the Corollary 3.1 with the convex edge cuts *T* and $T^{'}$, further studied according to the following cases:

**Case 1:** $$p \equiv 1 \mod 4$$

**Fig. 2:**
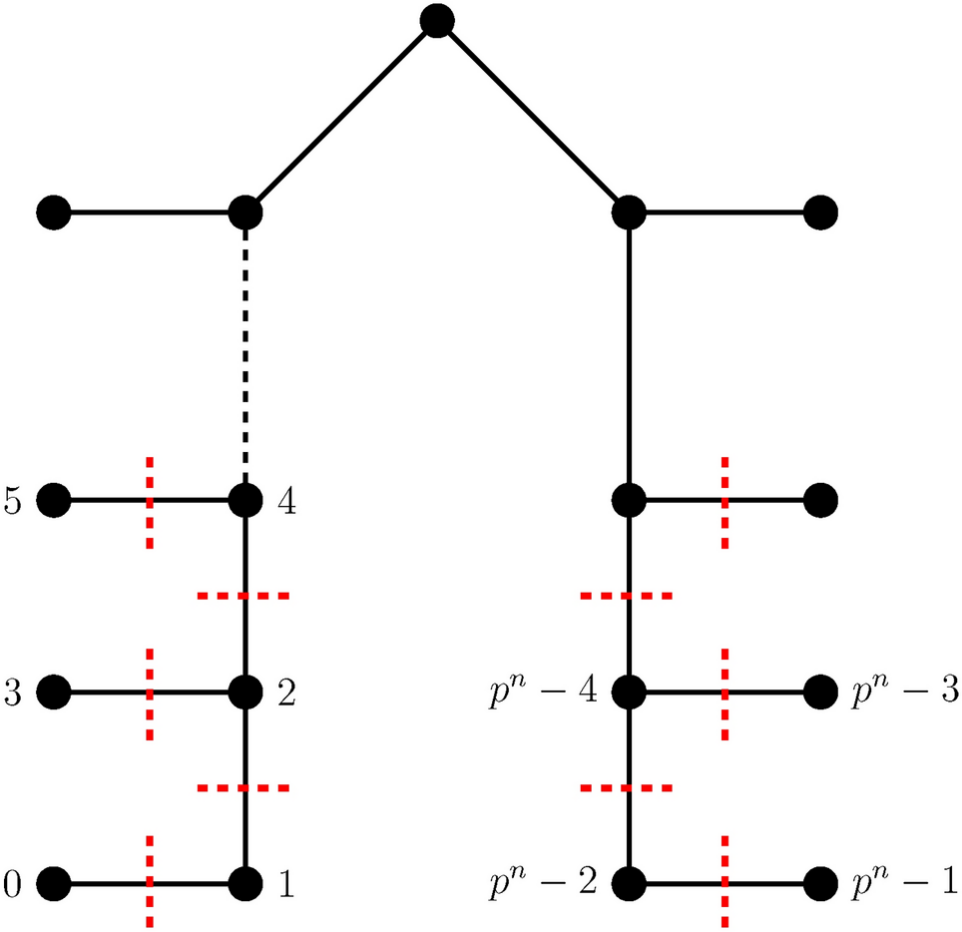
Example of arbitrarily fixed generalized banana tree as in case 1.

The graph $T(2, \left\lfloor \frac{p^{n} - 1}{4} \right\rfloor , \left\lfloor \frac{p^{n}-1}{4} \right\rfloor )$, takes the value $k = \left\lfloor \frac{p^{n}-1}{4} \right\rfloor$ for the case when $p \equiv 1 \mod 4$. The convex edge cuts that separate the graph into two parts are $T_{i}, 1 \le i \le \frac{p^{n}-1}{2}$ and $T_{j}^{'}, 1 \le j \le 2 \left\lfloor \frac{p^{n}-1}{4} \right\rfloor$ as $H^{1}_{T(2, \left\lfloor \frac{p^{n} - 1}{4} \right\rfloor , \left\lfloor \frac{p^{n}-1}{4} \right\rfloor )}$ and $H^{2}_{T(2, \left\lfloor \frac{p^{n} - 1}{4} \right\rfloor , \left\lfloor \frac{p^{n}-1}{4} \right\rfloor )}$ which are the inverse images of $G_{(K_{p}-C_{p})^{n}}^{1} = \Psi ^{-1}{\left( H^{1}_{T(2, \left\lfloor \frac{p^{n} - 1}{4} \right\rfloor , \left\lfloor \frac{p^{n}-1}{4} \right\rfloor )}\right) }$ and $G_{(K_{p}-C_{p})^{n}}^{2} = \Psi ^{-1}{\left( H^{2}_{T(2, \left\lfloor \frac{p^{n} - 1}{4} \right\rfloor , \left\lfloor \frac{p^{n}-1}{4} \right\rfloor )}\right) }$, that is represented in Fig. 2.

Thus $T_{i}$ and $T_{j}^{'}$ satisfy the conditions as in Lemma 2.1 thus inducing the optimal order that contributes to determine the edge congestions $E(T_{i})$, $1 \le i \le \frac{p^{n}-1}{2}$ and $E(T_{j}^{'})$, $1 \le j \le 2 \left\lfloor \frac{p^{n}-1}{4} \right\rfloor$ respectively. The minimum edge congestion by Lemma 2.1 is given by,

15 $$\begin{aligned} E(T_{i})= & n(p-3)\left( \frac{p^{n}-1}{2}\right) \end{aligned}$$

16 $$\begin{aligned} E(T_{j}^{'})= & \sum _{j_{1}=1}^{\frac{p^{n}-1}{4}}\Big ( 2nj_{1}(p-3) - 2I(2j_{1})\Big ) + \sum _{j_{2}=\frac{p^{n}+3}{4}}^{\frac{p^{n}-1}{2}} \Big (n(2j_{2}-1)(p-3) - 2I(2j_{2}-1) \Big ) \end{aligned}$$

Thus Eqs. 15, 16 and Lemma 2.2 results in the minimum wirelength for embedding $(K_{p}-C_{p})^{n}$ into $T(2, \left\lfloor \frac{p^{n} - 1}{4} \right\rfloor , \left\lfloor \frac{p^{n}-1}{4} \right\rfloor )$, where *p* is odd and $p \ge 9$ for $n\ge 2$, is given by

17 $$\begin{aligned} WL((K_{p}-C_{p})^{n}, T(2, \left\lfloor \frac{p^{n} - 1}{4} \right\rfloor , \left\lfloor \frac{p^{n}-1}{4} \right\rfloor ) )&= \sum _{i = 1}^{\frac{p^{n}-1}{2}} EC_{\Psi }(T_{i}) + \sum _{j = 1}^{ 2 \left\lfloor \frac{p^{n}-1}{4} \right\rfloor } EC_{\Psi }(T_{j}^{'}) \nonumber \\&=\left( n(p-3) \left( \frac{p^{n}-1}{2} \right) \right) + \nonumber \\&\sum _{j_{1}=1}^{\frac{p^{n}-1}{4}}\big ( 2n(p-3)j_{1}- 2I(2j_{1})\big ) + \nonumber \\&\sum _{j_{2}=\frac{p^{n}+3}{4}}^{\frac{p^{n}-1}{2}} \big ( n(p-3)(2j_{2}-1) - 2I(2j_{2}-1)\big ) \end{aligned}$$

Thus the minimum wirelength of embedding $\Psi : (K_{p}-C_{p})^{n} \rightarrow T(2, \left\lfloor \frac{p^{n} - 1}{4} \right\rfloor , \left\lfloor \frac{p^{n}-1}{4} \right\rfloor )$, is proved as given in Eq. 17.

**Case 2a:** $p \equiv 3 \mod 4$ **when** *n* **is odd**

**Fig. 3:**
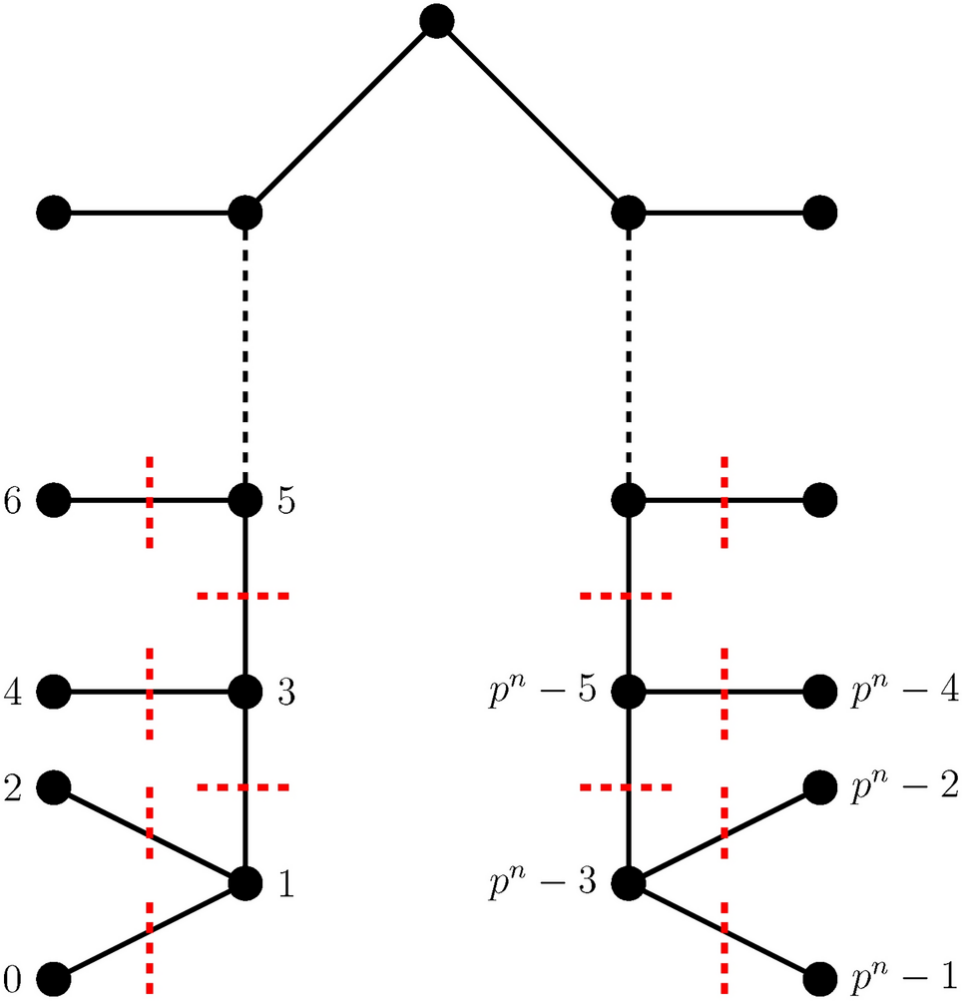
Example of arbitrarily fixed generalized banana tree as in case 2a.

The value of $k = \left\lfloor \frac{p^{n}-1}{4} \right\rfloor + 1$, when $p \equiv 3\mod 4$ hence $T(2, \left\lfloor \frac{p^{n} - 1}{4} \right\rfloor , \left\lfloor \frac{p^{n}-1}{4} \right\rfloor +1)$. $T_{i}$, $1 \le i \le 2 \lfloor \frac{p^{n}-1}{4}\rfloor +2$ and $T_{j}^{'}, 1 \le j \le 2 \left\lfloor \frac{p^{n}-1}{4} \right\rfloor$ partition the graph $(K_{p}-C_{p})^{n}$ into two disjoint sets by the definition of the convex edge cuts as represented in Fig. 3. The two components formed by these convex edge cuts are, $H^{1}_{T(2, \left\lfloor \frac{p^{n} - 1}{4} \right\rfloor , \left\lfloor \frac{p^{n}-1}{4} \right\rfloor + 1)}$ and $H^{2}_{T(2, \left\lfloor \frac{p^{n} - 1}{4} \right\rfloor , \left\lfloor \frac{p^{n}-1}{4} \right\rfloor + 1)}$, which are the inverse images of $G_{(K_{p}-C_{p})^{n}}^{1}$ and $G_{(K_{p}-C_{p})^{n}}^{2}$ respectively. The optimal order aids to determine the edge congestion $E(T_{i})$, $1 \le i \le 2\lfloor \frac{p^{n}-1}{4} \rfloor +2$ and $E(T_{j}^{'})$, $1 \le j \le 2 \left\lfloor \frac{p^{n}-1}{4} \right\rfloor$ is thus induced by $T_{i}$ and $T_{j}^{'}$ satisfying the conditions as in Lemma 2.1 respectively. The minimum edge congestion by Lemma 2.1 is given by,

18 $$\begin{aligned} E(T_{i})= & 2n(p-3) \Big (\left( \lfloor \frac{p^{n}-1}{4} \rfloor +1 \right) \Big ) \end{aligned}$$

19 $$\begin{aligned} E(T_{j}^{'})= & \sum _{j_{1}=1}^{\lfloor \frac{p^{n}-1}{4}\rfloor }\Big ( n(p-3)(2j_{1}+1) - 2I(2j_{1}+1)\Big ) + \sum _{j_{2}= \lfloor \frac{p^{n}+3}{4} \rfloor }^{\frac{p^{n}-3}{2}} \Big (2j_{2}n(p-3) - 2I(2j_{2}) \Big ) \end{aligned}$$

Thus Eqs. 18, 19 and Lemma 2.2 results in the minimum wirelength for embedding $(K_{p}-C_{p})^{n}$ into $T(2, \left\lfloor \frac{p^{n} - 1}{4} \right\rfloor , \left\lfloor \frac{p^{n}-1}{4} \right\rfloor + 1)$, where *p* is odd and $p \ge 9$ for $n\ge 2$, as given by

20 $$\begin{aligned} WL((K_{p}-C_{p})^{n}, T(2, \left\lfloor \frac{p^{n} - 1}{4} \right\rfloor , \left\lfloor \frac{p^{n}-1}{4} \right\rfloor + 1) )&= \sum _{i = 1}^{2\lfloor \frac{p^{n}-1}{4} \rfloor +2} EC_{\Psi }(T_{i}) + \sum _{j = 1}^{ 2 \left\lfloor \frac{p^{n}-1}{4} \right\rfloor } EC_{\Psi }(T_{j}^{'})\nonumber \\&= \left( 2n(p-3) \left( \left\lfloor \frac{p^{n}-1}{4}\right\rfloor + 1 \right) \right) \nonumber \\&+ \sum _{j_1=1}^{\left\lfloor \frac{p^{n}-1}{4} \right\rfloor } \big ( n(p-3) (2j_{1}+1) - 2I(2j_{1}+1) \big ) \nonumber \\&+ \sum _{j_{2}= \left\lfloor \frac{p^{n} + 3}{4} \right\rfloor }^{\frac{p^{n}-3}{2}} \big ( 2nj_{2}(p-3) - 2I(2j_{2}) \big ) \end{aligned}$$

Thus the minimum wirelength of embedding $\Psi : (K_{p}-C_{p})^{n} \rightarrow T(2, \left\lfloor \frac{p^{n} - 1}{4} \right\rfloor , \left\lfloor \frac{p^{n}-1}{4} \right\rfloor + 1)$, is proved as given in Eq. 20.

**Case 2b:** $p \equiv 3 \mod 4$ **when** *n* **is even**

This particular case follows Case 1 and thus has the same wirelength. $\square$

**Algorithm 5:**
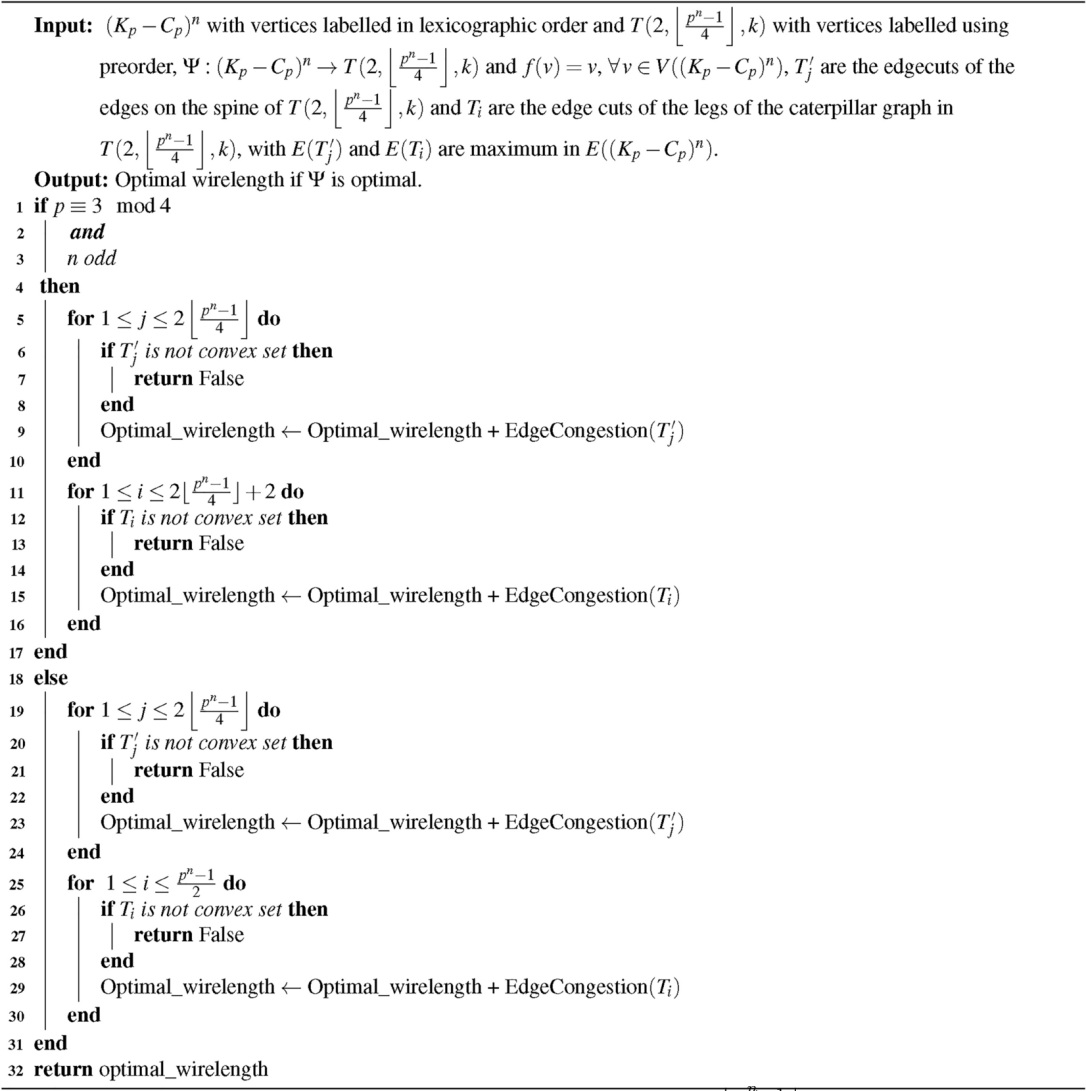
Optimal wirelength for embedding $(K_{p}-C_{p})^{n}$ into $T(2, \left\lfloor \frac{p^{n}-1}{4}\right\rfloor , k)$

### Time complexity of the algorithm

**Input:** $(K_{p}-C_{p})^{n}$ and Arbitrarily Fixed Generalized Banana Tree $T(2, \left\lfloor \frac{p^{n}-1}{4}\right\rfloor , k)$ where *p* is odd, $p \ge 9$ and $n \ge 1$ with $p^{n}$ vertices.

**Algorithm: ** The embedding algorithm 5.

**Output: ** The duration required to execute the embedding algorithm 5.

**Method: ** The time complexity of the algorithm is influenced by several factors; optimally ordering the vertices, denoted as $X$, identifying the convex edge cuts, calculating the minimum edge congestion, and determining the minimum wirelength of the embedding $\Psi$. There are two distinct cases to consider: one when $p \equiv 1 \mod 4$ and the other when $p \equiv 3 \mod 4$, specifically when $n$ is even,

$$\begin{aligned}&= p^{n} + 1 + 2\left( 2 \left\lfloor \frac{p^{n}-1}{4}\right\rfloor + \frac{p^{n}-1}{2} \right) \\&\le p^{n} \end{aligned}$$

When $p \equiv 3 \mod 4$, and *n* is odd

$$\begin{aligned}&= p^{n} + 5 + 8 \left\lfloor \frac{p^{n}-1}{4}\right\rfloor \\&\le p^{n} \end{aligned}$$

Therefore, the time required to compute the minimum wirelength for embedding $(K_p - C_p)^n$ into an Arbitrarily Fixed Generalized Banana Tree is $O(X)$, which is linear in complexity.

## Conclusion

We have identified the optimal layout for embedding $(K_p - C_p)^n$ into a grid structure and generalized book graph, emphasizing its critical role in the physical design of VLSI circuits. Furthermore, for non-linear data structures like the extended banana tree and the arbitrarily fixed generalized banana tree, we have also determined the optimal layout for the triangular snake. By advancing both the theoretical and practical aspects of graph theory and interconnection network design, our work opens opportunity for further research and applications in parallel domains. We conclude that the findings of this study will also have a significant impact on parallel computing systems.

## Data Availability

No datasets were generated or analyzed during the current study.
